# Development of a Chronic Care Model for Neurological Conditions (CCM-NC)

**DOI:** 10.1186/1472-6963-14-409

**Published:** 2014-09-19

**Authors:** Susan B Jaglal, Sara J T Guilcher, Tarik Bereket, Mae Kwan, Sarah Munce, James Conklin, Joan Versnel, Tanya Packer, Molly Verrier, Connie Marras, Kristen B Pitzul, Richard Riopelle

**Affiliations:** Department of Physical Therapy, University of Toronto, Toronto, Canada; Canadian Institute for Health Information, Toronto, Canada; Institute of Health Policy, Management and Evaluation, University of Toronto, Toronto, Canada; Department of Applied Human Sciences, Concordia University, Montréal, Canada; School of Occupational Therapy, Dalhousie University, Halifax, Canada; Morton and Gloria Shulman Movement Disorders Centre, Toronto Western Hospital, Toronto, Canada; Department of Neurology and Neurosurgery, McGill University, Montréal, Canada

## Abstract

**Background:**

Persons with neurological conditions and their families face a number of challenges with the provision of health and community-based services. The purpose of this study was to understand the existing health and community service needs and gaps in care and to use this information to develop a model to specify factors and processes that may improve the quality of care and health and well-being for persons with neurological conditions.

**Methods:**

We conducted semi-structured interviews with health care professionals, community-based non-health care professionals working with individuals with neurological conditions, and policy makers –from the Ministries of Health, Community and Social Services, Transportation and Education– across Canada. We used a purposive sampling and snowballing approach to obtain maximum variation across professions, sector and geography (provinces and territories, rural and urban). Data analysis was an iterative, constant comparative process involving descriptive and interpretive analyses and was initially guided by the components of the Expanded Chronic Care Model.

**Results:**

A total of 180 individuals completed the interviews: 39% (n = 70) health care professionals, 47% (n = 85) community-based non-health care professionals, and 14% (n = 25) policy makers. Based on the data we developed the Chronic Care Model for Neurological Conditions (CCM-NC). The major needs/gaps are represented by the following themes: acceptance and openness to neurological conditions, evidence informed policy, investments and funding, supported transitions, caregiver support, and life enhancing resources (education, employment, housing and transportation), knowledge and awareness of neurological conditions and availability and access to health services. The model maintains that intersectoral collaboration across the health system, community and policy components is needed. It recognizes that attitudes, policies, enhanced community integration and health system changes are needed to develop activated patients and families, proactive service delivery teams, a person-centred health system and healthy public policy for persons with neurological conditions.

**Conclusion:**

The CCM-NC will generate debate and discussion about the actions needed in each of the model components to enable people with neurological conditions to sustain healthier lives. Next steps include validating the model with persons with neurological conditions, in and outside of the Canadian context and developing and evaluating interventions to test the model.

**Electronic supplementary material:**

The online version of this article (doi:10.1186/1472-6963-14-409) contains supplementary material, which is available to authorized users.

## Background

According to the World Health Organization, neurological disorders and their sequelae are estimated to affect as many as a billion people worldwide [1]. They affect individuals across the lifespan and include childhood conditions such as cerebral palsy, Tourette syndrome or epilepsy; multiple sclerosis in early adulthood; and Parkinson’s disease or Alzheimer’s disease in late adulthood. In Canada, neurological disorders will become the leading cause of death and disability within the next 20 years [2]. The challenges of accurate diagnosis, health care provision including rehabilitation and ongoing management have a profound impact on individuals, their families and communities, as well as the overall health system and governments. There is little information on how to address these challenges, highlighting the need for an evidence-based multi-system level model that captures this complexity. This led the Canadian government in 2009 to fund the National Population Health Study of Neurological Conditions (NPHSNC), which is an extensive research program to inform future program and policy development comprising three national surveys and 13 research projects [3]. The study presented in this article is one of these research projects.

The aim of the present study was to identify needs and gaps in health and other related community-based services from the perspective of service providers and policy-makers, and use this information to develop a model to specify factors and processes that may improve the quality of care and health and well-being for persons with neurological conditions.

Four main underlying principles guided this work. First, while the bodies of knowledge and expertise vary for each condition, the system design pre-requisites are similar as each distinct neurological condition depends on the same large and complex health and social system. Second, neurological conditions create a significant and often catastrophic impact on individuals, their families and caregivers, and this creates needs and gaps that extend beyond physical and mental health. Third, the model developed must be consistent with Article 13 of the 2006 UN Convention on the Rights of Persons with Disabilities, which asserts the obligation of states ‘to ensure to persons with disabilities access, on an equal basis with others, to the physical environment, transportation, information and communications’ [4]. And fourth, the chronic nature of these conditions makes them amenable to the core principles of the Chronic Care Model (CCM) [5–8] and the Expanded Chronic Care Model (Expanded CCM) as they emphasize the need for an integrated system that not only addresses physiological and psychological recovery, but also enables individuals to take an active part in managing their own condition [9].

The CCM is an organizing framework for improving chronic illness care that incorporates patient, provider, and system level factors and interventions. Key components of the CCM such as self-management support, delivery system redesign and decision support contribute to more productive interactions with health care teams resulting in better health outcomes for individuals [10–12]. Thirty-two of 39 studies have reported that interventions based on CCM components improved at least one process or outcome measure for persons with diabetes, and 18 of 27 studies have demonstrated reduced health care costs or lower use of health care services [13].

Many jurisdictions across Canada have adopted the Expanded CCM as a blueprint for health system reform [9]. The main difference between the CCM and the Expanded CCM is the latter embeds the health system in a broader “community” context by integrating population health promotion principles to address health determinants. It is believed that changes in the community and health system will cumulatively lead to a partnership of informed, activated patients and prepared and pro-active health care teams that will work together to achieve better health outcomes [11]. In addition, more holistic models which consider a person’s family, social and political context are needed. Using the Expanded CCM as a guiding framework this article presents the development of the “Chronic Care Model for Neurological Conditions (CCM-NC)”.

## Methods

### Study design

This is a descriptive qualitative study. We conducted semi-structured interviews with health care providers, administrators and policy makers to understand 1) the existing health and community service needs and gaps in care for individuals with neurological conditions and their family members/caregivers; and, 2) the perceived health system level facilitators and barriers to management. Based on the NPHSNC, the *priority neurological conditions* included: Amyotrophic lateral sclerosis (ALS), Alzheimer’s disease and related dementia, brain tumours, cerebral palsy, dystonia, epilepsy, Huntington’s disease, hydrocephalus, neurotrauma (including brain and spinal cord injuries), multiple sclerosis, muscular dystrophy, Parkinson’s disease, Rett syndrome, spina bifida and Tourette syndrome. Approval for this study was obtained from the University of Toronto Research Ethics Board, Health Canada and Public Health Agency of Canada, Dalhousie University, Concordia University, and the Health Research Ethics Authority (Newfoundland and Labrador). This study adheres to the RATS guidelines (http://www.biomedcentral.com/authors/rats) for reporting qualitative studies.

### Development of interview guide

We constituted a Stakeholder Advisory Group (SAG), which included 18 members representing the different Neurological Health Charities of Canada member organizations of the NPHSNC. Initial interview questions were developed by the research team. Telephone consultations with each SAG member were conducted to assist in the development and refinement of the interview questions and associated probes. The interview guide was pilot tested with five SAG members regarding overall clarity, relevance, and specific wording and piloted twice in French. Finally, members of the research team reviewed two pilot interview transcripts, where suggestions were obtained for adding further probes and fine-tuning questions (See the ‘List of interview questions’ section).

### List of interview questions

List of open-ended questions from interview guide:

Overall Experiences

- From your own experiences, what are your overall thoughts about current services for people with [specific neurological condition] and those involved in their care giving?

Best Practices/Exemplary Programs

- What best practices facilitate better service provision for people with [specific neurological condition] and those involved in their care giving? (locally/provincially/federally)

Gaps & Needs

- What are some of the existing barriers that limit/impact the services for people with [specific neurological condition] and those involved in their care giving? (locally/provincially/federally)

Transitions

- Some people have brought up issues related to transitions (e.g. see probes below); do you have any comments related to transitions?

Current Policies

- What are your thoughts on the current policies related to the delivery of services to support people with [specific neurological conditions]? (locally/provincially/federally)
- What policy development or other activities could improve services for people with [specific neurological condition] and those involved in their care giving? (locally/provincially/federally)

Recommendations

- What strategies would address the support systems for people with [specific neurological condition] and those involved in their care giving? (locally/provincially/federally)

(Support system = family in general, informal caregiver, nursing home staff, etc.)

- In your opinion, what practical supports and/or small changes could be introduced that could have huge impacts in the long run? (locally/provincially/federally)
- What larger scale initiatives could be introduced that might address gaps in the delivery of services/support for people with [specific neurological condition] and those involved in their care giving? (locally/provincially/federally)

Technologies and Innovations

- Where would potential investment(s) be best directed to maximize impact of programs/service delivery /supports for people with [specific neurological condition] and those involved in their care giving? (locally/provincially/federally)
- Which strategies are best developed at the organizational level that requires further support and investment from other players such as government, policy makers, and funders?

(Organizational level = organization affiliated with and/or organizations in contact with)

Final Thoughts

- Do you have any final thoughts that you’d like to add that could inform our study? In particular, are there any unique factors/conditions that need to be addressed as it pertains to [specific neurological condition]?

### Sampling and data collection

The target population consisted of three stakeholder groups: health care professionals (HCPs), community-based non-health care professionals (NHCPs) and policy makers (PMs). We used a purposive sampling and snowballing approach to obtain maximum variation in perspectives across professions, sector and geography (provinces and territories, rural and urban) [14]. We reviewed websites from relevant ministries, regional health authorities and community organizations for individuals that would be knowledgeable about services, program planning or funding for persons with disabilities from each of the 10 provinces and 3 territories. Additionally, SAG members provided names of local opinion leaders (physicians, rehabilitation professionals, and psychologists) knowledgeable about the needs of individuals with neurological conditions. A total of 842 potential key informants were identified.

We aimed to have between 15–20 interviews [15] for each of the 13 provinces and territories, to obtain comprehensive perspectives on the 16 neurological conditions among the different stakeholders. It was important to have sufficient interviews from each province and territory because in Canada even though there is a universal health care system, health is under provincial jurisdiction and each province and territory operates its own health insurance plan. To obtain maximum variation [14], we estimated that 180 individuals would need to be recruited. Following each interview we cross checked our sampling frame against completed interviews to determine the type of informant to subsequently recruit. All interviews were conducted by telephone in English or French. Informed consent was obtained at the time of the interview. All interviews were digitally recorded and transcribed verbatim. Following each interview, research staff wrote detailed reflexive notes on ideas and emerging themes that were later discussed in detail with the broader team.

### Data analysis

The theoretical approach underlying this qualitative study was that of relativist ontology where a priori knowledge helped inform assumptions but allowed for emerging themes to arise [14]. Data analysis was an iterative constant comparative process involving descriptive and interpretive analyses [14, 16, 17]. Using the template analysis approach [18], a flexible coding structure was initially developed based on the major components of the Expanded CCM (self-management/develop personal skills, delivery system design/re-orient health services, decision support, information systems, build healthy public policy, create supportive environments; and strengthen community action) [9]. This allowed for creation of additional or “free” nodes when new emerging ideas or themes were identified. Data management was facilitated using NVivo 9 [19]. Two primary analysts (TB and MK) coded the transcripts according to standard qualitative coding techniques for thematic analysis [15] and “inter-coder” agreement [20] was established by cross checking 10 independently coded transcripts.

Pairs of research team members were assigned two components and asked to tabulate critical quotations and create a summary template highlighting the main themes. Nodes that emerged but did not fit the Expanded CCM components were noted and assigned to new categories. To the pre-determined list we added two new components: ‘Mobilizing Technology’ and ‘Knowledge Building & Educational Initiatives’. There were many instances when new themes were identified and existing components were not as prevalent in the data which prompted a second level of analysis. Four research team members (SJ, SJT, MK, TB) recoded and organized the themes to develop a model that better reflected the current needs of service providers and persons with neurological conditions.

## Results

From January to October 2012, invitations were sent to 338 of the 842 potential key informants: 89 did not respond, 69 declined and 180 interviews were successfully completed (39% (n = 70) were HCPs, 47% (n = 85) were NHCPs, and 14% (n = 25) were PMs). Interviews were conducted in either English (n = 171) or French (n = 9), based on the participants’ preference. On average, the interviews took approximately 45 minutes to complete. Table 1 specifies the distribution of types of service providers and PMs interviewed from each province/territory, inclusive of service catchment areas.

**Table 1:** **Distribution of key informants interviewed by type of neurological condition represented by province/territory**

	Neurological conditions (n = 16)																
Province/Territory	Acquired brain injury	Amyotrophic lateral sclerosis	Alzheimer’s and/or related dementia	Brain tumour	Cerebral palsy	Dystonia	Epilepsy	Huntington disease	Hydrocephalus	Spinal cord injury	Multiple sclerosis	Muscular dystrophy	Parkinson’s disease	Spina bifida	Rett syndrome	Tourette syndrome	Inclusive of all neurological conditions
**Nova Scotia**		▪	▪	▪	☆	☆		▪		☆	☆	▪☆		▪☆		☆	▪ ▿
**New Brunswick**		▪	▪	☆	▪	☆	☆	▪☆		☆	▪	▪☆	☆	☆			▿
**Prince Edward Island**		▪☆	☆					▪		☆		▪☆	☆				▪ ▿
**Newfoundland & Labrador**	☆	☆	☆			☆		▪			☆	▪▿☆	▪	▪		▪☆	▪▿☆
**Ontario**	▪☆	☆	☆	▪☆	▪	☆	☆	▪	▪	▪☆	☆	▪ ▿☆	☆		▪	▪☆	▪▿
**Quebec**	☆		▪☆		☆	☆	▪☆	☆		☆		▪☆	▪	▪☆	☆	▪	
**Northwest Territories**	▪	☆							▪	▪	☆	▪		▪	▪		▿
**Nunavut**							☆		▪	▪	☆	☆			▪		▿▪☆
**Yukon Territory**	☆	☆			▪		☆		▪	▪		▪			▪		▪ ▿
**Alberta**	▪☆	☆	☆		▪	▪☆	☆		▪	▪	☆	▪☆	▪		▪		▪ ▿☆
**British Columbia**	▪☆	☆	▪ ▿	▪☆	▪☆		☆	☆	▪	▪☆	☆	▪☆	☆	▪	▪		
**Manitoba**	▪☆	☆	▿	▪	☆	▪		☆	▪	▪	☆	☆	▪☆		☆		▿
**Saskatchewan**	▿☆	☆	▪		▪☆		▪		▪	▪	☆	▪			▪☆		▿

Key: ☆ Non- Health Care Professionals.

▪ Health Care Professionals.

▿ Policy Maker.

### Components of the Chronic Care Model for Neurological Conditions (CCM-NC)

**Figure 1:**
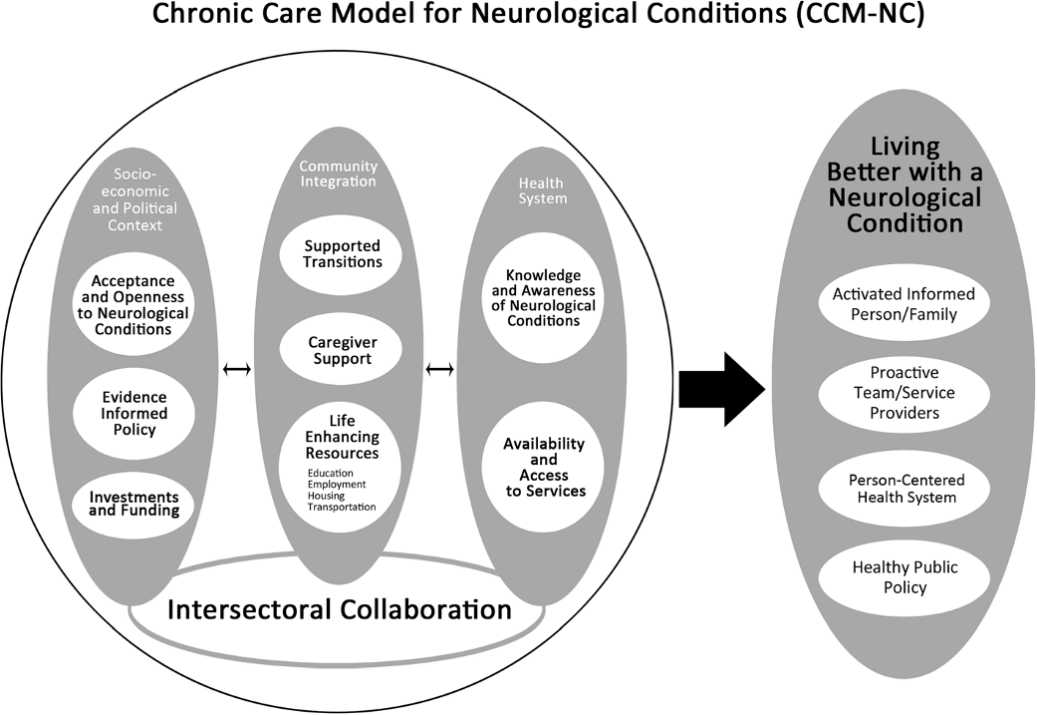
**Chronic Care Model for Neurological Conditions (CCM-NC).**

Ten themes emerged from our analyses and were organized into four groupings: the socio-economic and political context, community integration, the health system and intersectoral collaboration (See Figure 1). The following section describes the main themes or components of the model by group. Additional quotes supporting the identified themes can be found as an Additional file 1).

### Socioeconomic and political context

Three major themes emerged from the data that represented ways in which addressing needs and gaps related to the socioeconomic and political context could improve the quality of life for persons living with neurological conditions and their caregivers. These were: (i) acceptance and openness to neurological conditions, (ii) evidence-informed policy and, (iii) investments and funding.

#### Acceptance and openness to neurological conditions

Participants spoke about the negativity surrounding neurological conditions particularly related to behavioural disturbances, from the general public, those in schools, healthcare providers, employers. This negativity has led to stigma and marginalization resulting in feelings of exclusion and isolation, lack of acceptance of condition, and embarrassment. These poor attitudes have resulted in delays in diagnoses and treatment, less support from providers and fewer educational and employment opportunities and for those with the conditions can result in them not seeking out available services or being unaware of how to find help.

*It is so stigmatized that people don’t disclose that they have the condition. We do not even see the tip of the iceberg in our community organizations because if you can hide the fact that you have epilepsy, you will....They do not want to be identified as having seizures because they’re worried that some other human rights will be violated…. (Epilepsy, Community-based non-health care service provider, Opinion Leader, Ontario)*

#### Evidence informed policy

Participants noted gaps in current policies and a lack of available policies. A number of issues were highlighted including the need for policies to be: (1) reflective and supportive of persons with neurological conditions, (2) needs-based rather than condition specific and inclusive of lesser known neurological conditions, (3) encouraging of individual engagement and autonomy; (4) supportive of seamless transitions and integration into the community; and, (5) uniform and standardized across provinces. Another issue noted was the lack of awareness and knowledge about available policies among service providers.

*The role of the federal government in the development of the policy around disabilities is becoming less and less. It’s seen more as something that falls within the jurisdiction exclusively of the province and territories which mean there is precious little federal national leadership…. We still nationally as well as within all provinces and territories have a policy framework that does more to exclude than it does to include persons with disabilities. Despite Canada’s signing of the ratification of the UN convention on the rights with persons with disabilities… we are still a long ways from being in full and absolute compliance with that document… (Representing all neurological conditions, Disabilities Advocate, Newfoundland and Labrador)*

#### Investments and funding

Participants highlighted a number of areas where investments should be made. Many identified the need to 1) enhance health and community-based services and programs; for example, mobilizing technology with telehealth, medical and equipment programs, investing in mental health programs, homecare services, caregiver support and transportation; and, 2) support training for staff and caregivers to increase human resource capacity. Two other sub-themes addressed issues with current funding and insurance coverage. Participants talked about the inconsistency in funding particularly for school aged children. They highlighted the rigidity of eligibility criteria and the difficulty identifying appropriate funding sources, particularly noting how the time spent to complete applications for funding caused delays for patients. Many participants commented on inequities between private and public insurance coverage. With respect to obtaining private insurance, discriminatory practices included increased fees that discouraged persons from disclosing conditions and also the inability to secure personal insurance coverage.

*Private insurance companies…if you have a child with a disability, a lot of times you’re automatically disqualified from receiving insurance. Luckily, if they’re working, your child is covered until they’re 18 or 20 or 23…. But what happens when the client is at the end of their family’s insurance, can’t get it on their own and has like minimal employment? A lot of times that’s when they end up on community services because that is the only step for them. (Spina Bifida, Community-based non-health care service provider, Community Organization Representative, Nova Scotia)*

### Community integration

#### Supported transitions

Transitions between hospital, community and long-term care institutions are common for the neurological population and these transitions are exacerbated by co-morbid psychological and physical conditions. Unique to this population compared to other chronic conditions is the high prevalence among children. As a result, a major theme identified by participants was the “transition cliff” when children move to adulthood. They commented that transitions were not a positive experience as there was a lack of continuity of care within and between health sectors and government departments, lack of connectivity of information and services and lack of inclusion of family doctors. The lack of support for post-secondary education or employment further marginalized these youth. Other transition gaps highlighted were between inpatient care and community services where there is lack of access to exercise/rehabilitation programs not equipped to deal with the complexity of neurological conditions. Finally for those with dementia or ALS there was the need for case coordination, protocols and overall support for transitions to long-term care.

*So you have a 16 year old who’s probably only about 14 being forced into an adult facility where they don’t have the same kind of knowledge as they do in the pediatric world. They don’t have the same treatment modalities and so we call that a transition cliff and these kids literally fall off it. Their parents don’t know what to do…[in the adult system] the parents actually are not involved in their treatment or not allowed to be involved in their treatment. That’s a huge issue. (Tourette syndrome, Community-based non-health care service provider, Opinion Leader, Ontario)*

#### Caregiver support

Participants frequently highlighted the importance of informal caregiving and articulated the variety of roles that caregivers play. Informal caregivers are expected to function as case manager, service provider and advocate but have limited time available to be engaged in the family or friend role. Participants outlined a number of unmet needs for informal caregivers, such as training and education to prepare them for their supportive role and to help them to cope with behavioural challenges, promote autonomy and empowerment and to navigate the health care system. Support services needed were increased home support services, respite care options, opportunities for peer support and assistance with completing government forms,

*Often caregivers… because MS can be diagnosed so young and a lot of people have young children, the caregiver is trying to keep up with the house, do everything with the kids. If their partner is affected more severely, that’s a lot of burden and there’s a lot of caregiver stress and burnout that we see. I guess just more, say, supplemental support just to be able to take the odd break. That would be good. (Multiple Sclerosis, Community-based non-health care service provider, Community organization representative, Saskatchewan)*

#### Life enhancing resources (education, employment, housing and transportation)

We heard that the education system is not able to accommodate students as teachers and staff are not familiar with the impact of neurological conditions on students. Funding cuts have reduced classroom support workers. Participants identified a gap in meaningful and appropriate employment opportunities for both obtaining work (lack of accessible training programs) and returning to work. Discriminatory hiring practices add to the situation, contributing to high unemployment rates. Lack of appropriate housing was a major issue as participants stressed the need for affordable, accessible, safe and adaptive housing that is age specific. For example, younger individuals with neurological conditions are frequently placed in long-term care institutions because they cannot afford home modifications. Finally, availability and accessibility of transportation was seen as a key life enhancing resource to enable persons to attend medical appointments in a timely manner and participate in social activities outside of the home.

*These are young adults that need to be treated like young adults and not be treated like the elderly. It’s just changing people’s attitudes that they need a sense of belonging. They need a sense of stimulation. They just simply cannot sit in a group home staring at Dr. Phil all day. Like we have to put community programs in there and get them out in community doing whatever, daily activities. Once they’re done school, they’re pretty much out there staring at the walls either at home or in some kind of an institution. It’s like let’s focus on their young needs even though they could be cognitively impaired or nonverbal. It might not look like the wheels are turning because they can’t speak but often times the wheels are really turning. They’re very emotional beings and they need music and they need play therapy and they need human interaction. (Rett Syndrome, Health care professional, Registered Nurse, Saskatchewan)*

### Health system

#### Knowledge and awareness of neurological conditions

A significant lack of knowledge and awareness about neurological conditions among service providers was identified as a factor contributing to less than optimal care. In particular, the need for health care providers of persons with neurological conditions to acknowledge the potential episodic and chronic nature of neurological conditions was stressed.

*They were often kicked out of the hospital because the staff thought that they were drunk, but they were people with Huntington who actually needed help - but that wasn’t taken into consideration… (Huntington Disease, Community-based non-health care service provider, Community organization representative, Quebec)*

Information needs were widespread and extended across all of the priority neurological conditions. There was a desire for more knowledge about etiology, prevention, management, and services and supports available in the community. There was a specific need for increasing knowledge about acute behavioral disturbances as aggressive behaviour and poor motor control are misunderstood and mishandled. Primary care providers were identified as being aware of their lack of knowledge and frustrated by the system. Respondents wanted the development of more comprehensive evidence-based practice guidelines and online resources including webinars, Wiki platforms, peer-support, and innovative application software from credible sources. One of our most important findings was the lack of knowledge and understanding about self-management support among providers. Competencies related to use of self-management support strategies, protocols and interventions were called for. A particular need for self-management support was identified for youth and their parents who need to prepare for and manage the changes inherent in transition to the adult health care system.

#### Availability and access to health services

Participants highlighted the need for person-centred care requiring a shift from diagnosis to functional requirements and needs. Participants spoke of an increased need for multi-disciplinary clinics with team-based care to assist with diagnosis, rehabilitation and ongoing management. They identified the need for integrative case planning and services and supports in the community to facilitate a smooth transition from home to long-term care admission.

*They suddenly require long term care placement but there’s no planning for that, so then what? It’s hard to get enough services in place in a home to help them continue care at home and there’s no beds available in long term care because you know there was no planning towards that. So the system wasn’t aware that they needed it. So these crisis events are happening and that’s not an ideal for either the system or clients or families to be dealing with that type of situation. We hear about that a lot. (Alzheimer’s and/or related dementia, Health care professional, Registered Nurse, Saskatchewan)*

A central theme was the lack of availability of health and community-based services in rural areas, which can create significant health inequities. Participants acknowledged that the small numbers of persons with neurological conditions in rural areas can make it difficult to recruit and retain specialists and also maintain existing skill sets. Living in a rural area also places additional costs on families as they need to take more time off work to drive long distances to appointments or relocate to an urban centre. There is also lack of access to special schools such that students cannot access post-secondary education due to lack of availability of attendant care resulting in greater burden on parents who remain the only caregivers. Particularly disconcerting is the limited range of housing options because for those who need additional support the only choice is a long-term care facility. Participants provided a number of suggestions for increasing access to services in rural communities including having a care coordinator, providing mobile clinics, increasing use of telehealth and respite services.

### Intersectoral collaboration

To improve the lives of persons living with neurological conditions, intersectoral collaboration is needed within and between settings. From the interviews, we noted that the lack of intersectoral collaboration results in negative outcomes. For example, organizations were competing with each other for scarce resources, duplicating services and lacking leadership and resources for sharing knowledge and expertise about neurological conditions. There was a need for improved collaboration and cooperation with joint policies, communication and shared assessments between ministries of Health, Community and Social Services, Transportation and Education as well as between service providers. Within the health sector, there was a need for sharing electronic health records and aligning services across regions.

*There has been traditionally a very medical model used in working with people with disabilities and with people with chronic diseases. I think by working together with say like non-profit community services organization… I think can really be much more integrated and holistic in working with people to have better health outcomes…. In that whole coordination piece, I think involving all those stakeholders that we try to bring to the table, the different ministries, the doctors, and the community piece. It’s really investing in their work to really streamline and align what we are doing…. I think we can really cover that provincially if we really put down those barriers and those silos and work together. (Multiple Sclerosis, Community-based non-health care service provider, Community organization representative, Alberta)*

In summary, like the Expanded CCM, the goals of the CCM-NC are to have an activated and informed person and family, a proactive team of service providers, a person-centred health system and healthy public policy to achieve improved well-being and better health outcomes for persons with neurological conditions [11]. It is hypothesized that this can be achieved by intersectoral collaboration between the health system, the community and the socio-political environment. This has implications for interventions at the policy and practice level.

## Discussion

In this pan-Canadian study with 180 key informants that identified needs and gaps in health and other related community-based services from the perspective of service providers and policy-makers we developed the Chronic Care Model for Neurological Conditions or CCM-NC to highlight individual, provider and system level factors, processes and interventions that may be important to improving the quality of care and health and well-being of persons with neurological conditions.

What this study has revealed is whilst the Expanded CCM underpins the current health system; persons with neurological conditions may require emphasis on different model components or interventions to achieve optimal health outcomes. A mapping of the CCM-NC and the corresponding Expanded CCM components is provided in Table 2.

**Table 2:** **Mapping of CCM-NC components to Expanded CCM**

Components of CCM-NC	Corresponding expanded CCM component
**Socio-economic and political context**	**Community**
• Acceptance and Openness to NC	• Create Supportive Environment
• Investments and Funding	• Create Supportive Environment
o Enhance health and community services by mobilizing technology	
o Support training of HCP and caregivers	
• Evidence-informed Policy	• Build Healthy Public Policy
o Needs-based/Reflective of neurological conditions	
o Encourages independence	
o Supports seamless transitions	
o Standardized across provinces	
**Community integration**	**Community and health system**
• Supported Transitions across care settings and lifespan	• Create Supportive Environment and Delivery System Design
• Caregiver Support	• Strengthen Community Action
• Life enhancing Resources (Education, Employment, Housing, Transportation)	• Create Supportive Environment
**Health system**	**Health system**
• Knowledge and Awareness among HCP	• Decision Support and Information Systems
o Guidelines	
o Online resources	
o Training in self-management principles	
• Knowledge and Awareness among clients	• Self-management/Develop Personal Skills
o Self-management support for youth and parents	
• Availability and Access to services	• Delivery System Design
o Need for person-centred care	
o Integrative case planning for smooth transitions	
o Lack of services in rural areas	
**Intersectoral collaboration**	**Productive interactions and relationships**
• Activated and Informed Person/Family	• Informed Activated Patient
• Proactive Team of Service Providers	• Prepared Proactive Team
• Person-centred Health system	• Activated Community
• Healthy Public Policy Across Sectors	• Prepared Proactive Community Partners

In developing the CCM-NC our data showed that interventions to improve *knowledge and awareness of neurological conditions and availability and access to health services* are a prerequisite prior to implementation of the components associated with the health system (self-management, delivery system design, decision support and information systems)*.* For example self-management is defined as “the individual’s ability to manage the symptoms, treatment, physical and psychosocial consequences and lifestyle changes inherent in living with a chronic condition” [21]. A key feature of self-management support is providing patients with the tools and skills to manage their chronic disease by increasing their confidence (self-efficacy) to prevent and deal with disease-related problems. The goal of the self-management component in the CCM and the Expanded CCM is collaboration between an informed and activated patient and family and a coordinated or proactive team of providers [5–8]. Our results showed that for neurological conditions this is a challenge as providers reported that they lack basic knowledge and awareness about neurological conditions and the principles of self-management. In fact, providers equated self-management with self-advocacy. Until we address this knowledge gap self-management will not be seen as a health system priority.

The *availability and access to health services* component of the CCM-NC incorporates three health system components of the Expanded CCM; delivery system design, decision support and information systems. The CCM strongly advocates for the use of evidence-based practice guidelines to support best practices and decision-making. However, one of the challenges with many of the priority neurological conditions, particularly the less common conditions, is a lack of evidence-based guidelines or protocols. Therefore the CCM-NC has less emphasis on decision support and information systems but emphasizes the need to promote equitable care and optimal outcomes through the development of standardized referral criteria, protocols, care pathways and guidelines [22, 23].

A major difference between the CCM-NC and the Expanded CCM is the re-conceptualization of the three components representing community resources and policies (create supportive environments, build healthy public policy and strengthen community action). For persons living with neurological conditions, interventions to create a supportive environment are critical since a major goal is community integration. In the Expanded CCM, actions to create a supportive environment are described as generating living and employment conditions that are safe, stimulating, satisfying and enjoyable. For persons with a neurological condition, these are necessary but not sufficient because they do not fully capture two other areas critical for improved quality of life, namely *caregiver support* and *supported transitions*. Cognitive and physical impairments are common and therefore caregivers play an important role and are viewed as the secondary team [24]. Family caregivers are largely operating with little support from formal services, and with few educational opportunities [25]. The emotional costs and stress levels—including physical, psychological, social, and financial reported by family caregivers—are high, especially for those managing the physical demands of caring for individuals with a disability [25–32]. Support is particularly needed at times of transitions into different care settings. Two common and difficult situations are children who have to transition from the integrated paediatric system to the fragmented adult health care system [33–36] and adults from the community to a long-term care institution [37, 38]. Therefore implementation of interventions to support caregivers and transitions become increasingly important in strengthening community action and creating a supportive environment.

Another important theme of the CCM-NC is *acceptance and openness*. When compared to other chronic conditions, stigma is unique to neurological conditions. It is an important public health problem that increases the toll of illness as many individuals with neurological conditions will experience serious limitations to their enjoyment of economic, social and cultural rights and have many unmet needs in the areas of civil rights, education, employment, residential and community services, and access to appropriate health care [1, 39, 40]. Fighting stigma and discrimination requires education of health care providers and the public in order to dispel common myths and promote positive attitudes to create a supportive environment and build healthy public policy [1, 41].

Finally, the findings of this study have major implications for policy and practice. Using the CCM-NC as a guide we have to change the policy mindset from short-term and restrictive to investing in the long term with more flexible policies. For example more flexibility in income support and employment benefits and increasing opportunities to participate in the workforce or in post-secondary education and training programs are needed. Policy-makers also have to create more opportunities for affordable and accessible housing that is age specific and appropriate to support independence. Many of these policies would involve different ministries including health, housing, education and employment and would benefit from the creation of shared assessments to improve linkages and cooperation. A more direct implication for practice is increasing knowledge and awareness of neurological conditions and availability and access to health services. This may require development of educational resources for health care professionals and persons with neurological conditions and their families. Whereas increased access to specialists and multidisciplinary care teams may require developing telehealth and health human resource planning to increase expertise in neurological conditions. One of the challenges of the model will be prioritizing these various initiatives, which may be different depending on the underlying health and social care system in a country.

### Strengths and limitations

Our approach has a number of strengths and limitations. One, face validity of the model was obtained through a meeting of the SAG and five webinars conducted with key informants across the country. Two, recruitment of key informants from across the country, settings and conditions increased the generalizability of the findings. Whereas, many other models have focused on single neurological conditions or only one aspect of care, for example community-based rehabilitation for ABI [42], an integrated model of Alzheimer’s disease care in a primary care setting [40, 43], or community-based rehabilitation model for Parkinson’s disease [44] or are opinion pieces without empirical data [44, 45]. Three, in support of the development of the CCM-NC, a recent position paper by Corrigan and Hammond on addressing traumatic brain injury states that we need to consider a chronic disease management approach to reduce costs and improve quality of life [46]. The main limitation of the CCM-NC is that individuals with neurological conditions were not interviewed. However, input was received from health and community-based service providers, advocacy groups and the Neurological Health Charities of Canada member organizations.

## Conclusions

The intent is that the CCM-NC will generate debate and discussion about the changes needed in policy and practice. Next steps include validating the model in persons with neurological conditions and outside of the Canadian context to assess the potential for international relevance especially in countries with a universal health care system. Future work will involve developing and evaluating interventions guided by the model to improve quality of care, health outcomes and well-being for individuals with neurological conditions.

## Electronic supplementary material

Additional file 1: **Representative Quotes for Needs and Gaps identified within the CCM-NC.** (DOCX 21 KB)
