# Supplementary material for: Development of a Chronic Care Model for Neurological Conditions (CCM-NC)
Source: BMC Health Serv Res. 2014 Sep 19;14:409. doi: 10.1186/1472-6963-14-409 (PMC4262116; doi:10.1186/1472-6963-14-409)
Supplement: Supplementary file 1 — Additional file 1: Representative Quotes for Needs and Gaps identified within the CCM-NC. (DOCX 21 KB) [file 12913_2014_3641_MOESM1_ESM.docx]

Additional File 1. Representative Quotes for Needs and Gaps identified within the CCM-NC

| **SOCIOECONOMIC AND POLITCAL CONTEXT** | **Acceptance and Openness to Neurological Conditions** | *On a provincial level I think it’s literally just a matter of changing mindset…. If we were just able to take brain injury out of the mental health diagnosis and out of the CP clinic, the same time would be used to treat the people but just under the right banner. The biggest policy issue is that they try to squeeze ABI into every other box. (Acquired Brain Injury, Community-based non-health care service provider, Newfoundland and Labrador)*  *Loneliness is a big thing, just trying to connect with people. I mean in [Name of city], especially in the winter, the weather does have some big barriers on getting out. So people tend to stay in their house and they get lonely and connecting people is hard. (Cerebral Palsy, Community-based non-health care service provider, Manitoba)* |
| --- | --- | --- |
|  | **Evidence Informed Policy** | *There tends to be this ‘oh no we’ll open the floodgates’ type of thing about the approach to writing policy. I think it’s a philosophical shift from minding the vault to investing in people and recognizing that policy really needs to be written to invest in people so that you reduce their need to rely on the system…. (Spinal Cord Injury, Health Care Professional, Organization, Yukon)*  *The problem is that healthcare is provincially legislated…. It’s health breakdown….For a country that is as wealthy as ours, our inability to help people and provide services is abysmal. It’s sad and tragic…. This whole concept of universal healthcare, it was to have access, right. Well the problem is that there’s no access. People that re providing care are overwhelmed… If they advocate… then they’re intimidated or bullied… Or protect themselves… turn a blind eye to it…. When things are fine everything is good and you can divvy it up however you want. But when things are not good, you need a captain to man the ship. (Rett Syndrome, Health Care Professional, Physician-Alberta)* |
|  | **Investments and Funding** | *Funding is a huge issue of course, as it is for every diagnosis, specifically funding for care in home and for equipment. In New Brunswick we only have funding really through social development for people who are very poor. If you are middle class, if you don’t have private insurance, you’re in a lot of trouble because you won’t have funding for equipment…. (Amyotrophic Lateral Sclerosis, Health Care Professional, Rehabilitation Centre, New Brunswick)*  *I’d put [funds] in the community. I’d be looking for those community services, those primary care doctors, family health units, family health networks that you could add some additional dollars that would engage them in this population. (Spinal Cord Injury, Community-based non-health care service provider, Opinion Leader, Ontario)*  *I guess just more resources to provide more programs in rural areas, more day programs, more respite, more case managers, more training, more access to ongoing continuing education programs, that kind of thing, more behavior management consultants. (Alzheimer's and/or related dementia, Health Care Professional, Registered Nurse-* *Saskatchewan*) |
| **COMMUNITY INTEGRATION** | **Supported**  **Transitions** | *I am not so sure that we’ve made it all that easy in our healthcare system for the care to be moved from the community into long term care and then if required from long term care into acute care and then back to long term care. Those I think are very important points of transition that have been largely ignored by the sector. (Alzheimer's and/or related dementia, Community-based non-health care service provider, Policy Advocator, Ontario)*  *They will focus on getting people physically to a point where they say they should be able to do sedentary work but then there’s no transition from that to finding an actual job....there’s a non-profit organization in PEI [(Prince Edward Island)] that helps people who are applying for CPP disability...job searching....they are filling in that gap where people are transitioning from coping with a disability to reintegrating into a competitive workforce. That is where our big gap is. (Spinal Cord Injury,* *Community-based non-health care service provider, Advocacy Lawyer, New Brunswick)* |
|  | **Caregiver Support** | *We expect families to take care of business and we give them no credit whatsoever and we give them no training. They are treated as invisible and not everyone can do the kind of thing that we ask them to do. (Brain Tumour*, *Health Care Professional, Social Worker, Manitoba)*  *We’ve had talks about that, having like a Facebook kind of system for patients so that they connect with each other or they can connect with other caregivers and organize care for a patient that way. We haven’t developed any but I know there are a lot of people who are looking at that, developing programs for chronic disease and helping caregivers manage a bit better. We don’t have anything like that in place. (Brain Tumour, Health Care Professional, Nurse, British Columbia)*  *I really do think that family caregivers need counseling. They need professional counseling, ethical counseling. The issues of being a caregiver and trying to sort out these transitions for their loved ones and trying to manage the stress of sudden changes and just how to… figure out how to make themselves really available to their parent. There are certain times when the caregiver’s life just completely goes on hold. They take a leave from work, they don’t see their husband for a while, and they go live in a different province. Like it’s just a major disruption for family caregivers and they need counseling and support, professional counseling. (Alzheimer's and/or related dementia, Health Care Professional, Saskatchewan)* |
|  | **Life Enhancing Resources:**  **Education**  **Employment**  **Housing**  **Transportation** | *The education system just like many other systems does not understand the impact of a diagnosis of a brain tumor on a child. They don’t understand how that affects the child physically, mentally, cognitively because they probably have never seen a child with a brain tumor. So they don’t know what to do with them. They don’t know where to put them in the system because a child with a brain tumor is a round peg and all the holes are square. They just don’t fit. It can be very frustrating. It’s exhausting to have to fight constantly for the rights of your child. (Brain Tumour, Community-based non-health care service provider, Association-Ontario)*  *Employment support groups is a big gap here. We simply do not have any vocational support for persons with disabilities at all… We have generic supports that pretty much any unemployed person can access. But those programs don’t have employees who are skilled in training persons with disabilities and dealing with the barriers that these persons might face in a workplace. (Representing all neurological conditions, Policy Maker,* *Nunavut)*  *I mean we really need to begin to work on housing options in the community or finding the funds to adapt someone’s home so that they can transition from rehab to home. We need to start working on that the day the injury happens. (Muscular Dystrophy, Community-based non-health care service provider, Opinion Leader, Alberta)*  *The challenge for them is transportation to their appointments [at] the clinic…. We do outreach clinics but we can only do so many of those because we can’t be travelling and seeing our regular caseload as well…. [There is] pretty much [no] transportation available for clients [if they want] to come in person. [W]e’ve had some patients transported by ambulance. That’s sort of an extreme case where they absolutely have to come here…. Within the [name of city] we have dial a bus or special transit but that’s only within the city limits…If you don’t have a personal vehicle to get here and you can’t be transferred into a vehicle, then you don’t get here. (Amyotrophic Lateral Sclerosis, Health Care Professional, Rehabilitation Centre, New Brunswick)* |
| **HEATH SYSTEM** | **Knowledge and Awareness of Neurological Conditions** | *There are huge gaps in the knowledge of clinicians as well as family doctors… All of our surveys across the country suggest that family physicians are never trained in seizures in medical school. Unless they’ve had professional development sessions, they refer to specialists. That becomes problematic in places like the Yukon that doesn’t have specialists. (Epilepsy, Community-based non-health care service provider, Opinion Leader, Ontario)*  *I didn’t know what was available to her. I didn’t even know her condition to be perfectly honest, as a doctor. This is not something we learn in medical school. (Rett Syndrome, Health Care Professional, Physician, Ontario)*  *Sometimes Huntington’s can look like something else. A lack of understanding can lead to an escalation. (Huntington Disease, Community-based non-health care service provider, Social Worker, Saskatchewan)*  *Are we training them to look after a young population with acute problems or are we training them to look after a population where it’s chronic disease and dementia and the majority of people they’re going to be dealing with are older and sicker. That training shouldn’t have to happen after they graduate and they get to the workplace. It’s really problematic. (Alzheimer's and/or related dementia, Community-based non-health care service provider, Policy Advocator, Ontario)* |
|  | **Availability and Access to Services** | *Well the people in rural areas are really behind the eight ball because in some cities there are no neurologists at all. So they’re forced to come to the two big cities, [Name of two Major Cities] which is where the hospital epilepsy clinics are. Like in the [Name of Rural Area] there is not one neurologist yet [Name of Rural City] and surrounding area there’s over 100,000 people now…. People in the Northwest Territories, the same thing. They generally have to fly in to [Name of Major City] because there are no neurologists or epilepsy specialists in the Northwest Territories. (Epilepsy, Community-based non-health care service provider, Association, Alberta)*  *I would say rural communities have limited range of housing available like assisted living. What we hear is that there’s not as much choice of housing between living at home independently and living in a long term care facility. We need more in the middle. I think that is a challenge in rural areas. (Alzheimer's and/or related dementia, Health Care Professional, Registered Nurse, Saskatchewan)*  *Sometimes smaller communities and difficult geographies that don’t have a lot of specialists have a lot of balancing community support....with videoconferencing [and] some of the telemedicine approaches, I think we will be able to link people better...but it can be a problem for people who do need to leave their local area to get a service. (Alzheimer's and/or related dementia, Health Care Professional, Physician*, *British Columbia)* |
| **INTERSECTORAL COLLABORATION** | **Socioeconomic and Political Context, Community Integration and**  **Health System** | *They require input from physicians, outreach nurses, dieticians, speech language therapists, occupational therapists and social workers because the illness hits them in their prime and there are usually spouses or children in the house, work to be addressed, benefits to be addressed. So they require input from a truly multidisciplinary team. It’s not the case here. It’s very piecemeal here. (Huntington Disease - Health Care Professional -Psychiatrist-Nova Scotia)*  *The other challenge too is in those instances where you can get people to the table from various sectors, whether it’s mental health, addictions, the justice system, acquired brain injury, community support services, if you can get them to the table, which is a lot of work, and get them to bend their admission criteria so that everybody can contribute a piece that creates a whole care plan, that’s where we tend to be most successful…. If any one of those players steps back, the whole care plan falls apart. (Acquired Brain Injury,* *Community-based non-health care service provider -Association-Ontario)*  *I’m very passionate about how do we work more inter-professionally. We are still very siloed as a province in terms of how our disciplines work together….I think moving to models of more inter-professional coordinated care and linking them with I guess the places that make sense for them. So in many cases that is their GP office, their community health centers… (Representing all neurological conditions -Health Care Provider-Rehabilitation Center-Nova Scotia)* |
